# Analysis of NK cell clones obtained using interleukin-2 and gene-modified K562 cells revealed the ability of “senescent” NK cells to lose CD57 expression and start expressing NKG2A

**DOI:** 10.1371/journal.pone.0208469

**Published:** 2018-12-05

**Authors:** Maria A. Streltsova, Sofya A. Erokhina, Leonid M. Kanevskiy, Dean A. Lee, William G. Telford, Alexander M. Sapozhnikov, Elena I. Kovalenko

**Affiliations:** 1 Laboratory of Cell Interactions, Department of Immunology, Shemyakin-Ovchinnikov Institute of Bioorganic Chemistry, Russian Academy of Science, Moscow, Russian Federation; 2 Center for Childhood Cancer and Blood Disorders, The Research Institute, Nationwide Children’s Hospital, Columbus, OH, United States of America; 3 Experimental Transplantation and Immunology Branch, National Cancer Institute, National Institutes of Health, Bethesda, MD, United States of America; Karolinska Institutet, SWEDEN

## Abstract

In this work, we analyzed the phenotype and growth of human NK cell clones obtained by the stimulation of individual NK cells with IL-2 and gene-modified K562 feeder cells expressing membrane-bound IL-21 (K562-mbIL21). We generated clones from NK cells at distinct differentiation and activation stages, determined by CD56, CD57 and HLA-DR expression levels. Less differentiated CD56^bright^ NK cell subsets showed higher cloning efficiency compared with more differentiated CD56^dim^ subsets, especially with the CD57^bright^ subset. However, clones from the CD56^dim^CD57^–^ subset lived longer on average than other subsets. Moreover, several clones with the highest cell numbers were derived from CD56^dim^CD57^–^HLA-DR^−^cells. Most of the clones including those derived from more differentiated CD56^dim^CD57^+/–^NKG2A^–^ NK cells showed a less-differentiated NKG2A^+^ phenotype. Also, CD57^–^ cells were frequently observed in clones derived from CD57^+^ NK cells suggesting the loss of CD57 during the cloning process. On the other hand, KIR surface expression once detected for a clone never disappeared entirely, confirming irreversibility of the KIR expression. In summary, we have demonstrated that in specific conditions terminally differentiated CD57^+^ human NK cells are able to acquire the CD57^–^ phenotype that was previously not observed and, thus, was considered impossible.

## Introduction

The phenotype of NK cells changes during differentiation and activation, forming subsets with various functionalities [1,2]. By now, it is known that significant phenotypic and functional differences exist between the two subsets of NK cells: CD56^bright^CD16^dim/–^and CD56^dim^CD16^+^ [3]. CD56^bright^ NK cells are considered to be less differentiated NK cells with weak cytotoxicity compared to CD56^dim^ NK cells [4,5]. CD56^bright^ NK cells, however, are better producers of interferon-γ (IFN-γ) in response to cytokine stimulation. In contrast, CD56^dim^ NK cells are capable of antibody-dependent cytotoxicity via CD16 receptor recognizing Fc fragments of IgG [2]. CD56^dim^ cells also produce IFN-γ, but rather in response to contact interactions than cytokine stimulation. Some of CD56^dim^ cells express on their surface CD57, a terminally sulfated carbohydrate epitope, which marks highly differentiated and senescent NK cells. CD57 expression was shown earlier to be associated with a gradual loss of proliferative capacity [1,6]. CD56^bright^ and CD56^dim^ NK cells can differ in their expression patterns of NKG2A, KIR and other surface markers during differentiation process [1,7] that leads to additional variations in their functional activity and response to stimuli. Although basic markers which expression is associated with NK cell differentiation are well known, precise data about patterns of their expression in various conditions of NK cell stimulation are limited. Besides, functional capabilities of NK cells depend greatly on their activation state. Surface expression of HLA-DR (a type of MHC class II molecules) is considered as a NK cell activation marker associated with increased IFN-γ production and elevated degranulation [8]. NK cells are of considerable interest for immunotherapy, since they manifest cytotoxic activity against virus-infected and tumor cells. For clinical purposes, NK cells need to be activated and expanded *ex vivo* in conditions providing sustainable production of NK cells with desired properties, which can expand in sufficient quantity. However, final NK cell products are often characterized by high variability in context of growth rate and cytolytic abilities. A significant variation in NK cell expansion between different donors was reported in earlier works [9–11]. The reason for this phenomenon is still unknown. One explanation may be variations in NK cell ratio in different people, as well as in their receptor expression profile and their response to various contact and soluble stimuli [12]. Another cause may be different proportions of NK subsets with certain features in donors. The issue of great interest is to reveal the relations between NK cell phenotypic state after isolation *ex vivo* and its capacity to expand and increase its functional capabilities *in vitro* for further application in immunotherapy. A study on an individual cell level of NK cell response patterns to selected stimulatory conditions may address this question.

In the current work NK cell cloning has been used to investigate the fate of an individual NK cell proliferating in response to a stimulus. We have chosen the combination of IL-2 and K562 feeder cells, genetically modified to express membrane-bound IL-21 and other surface-associated molecules (K562-mbIL21) as an initial stimulation for cloning [9]. These stimuli were shown to lead to the steady proliferation of activated NK cells for 6 weeks and significant cell expansion [9]. IL-2 used in this model at initial stage and for weekly clone restimulation is one of the powerful inducers of NK cell proliferation [13]. It also stimulates the differentiation of NK cells and increases their functional activity [14]. IL-21, a cytokine produced by adaptive immune cells, also has pleiotropic effects on NK cells. Although IL-21 itself does not directly stimulate NK cell proliferation, both co-stimulating and inhibitory effects of this cytokine on IL-2/IL-15-induced proliferation have been shown in several studies [12,15–17].

Clones were generated from NK cell subsets distinct in differentiation and activation stages defined on the basis of CD56, CD57 and HLA-DR expression. We compared progenitor cells from these subsets in terms of: 1) responsiveness to IL-2/K562-mbIL21 stimulation by clonal expansion; 2) survival of the obtained clones over time and capacity to generate the longest-lived clones; 3) the phenotypic characteristics of the obtained clones. Basing on the phenotypic analysis of the growing clones, we have found the ability of terminally differentiated NK cells to change, acquiring CD57^–^NKG2A^+^ phenotype, that is typical for less mature cells.

## Results

### Clone generation efficiency depends on differentiation stage of NK cells

**Fig 1:**
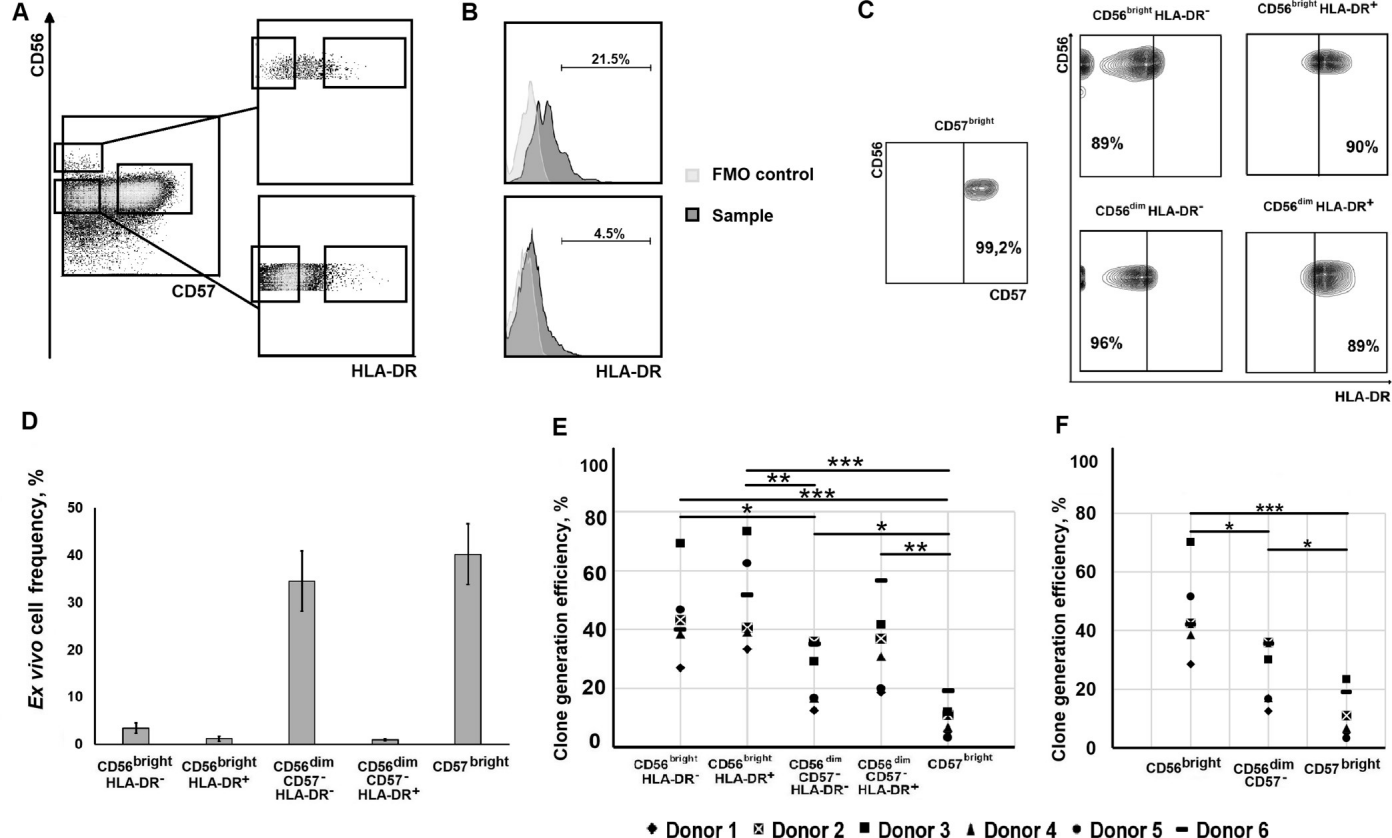
Comparison of the efficiency of clone formation in the presence of IL-2 and K562-mbIL21 of NK cells at different differentiation and activation stages. (A) Scheme of NK cell sorting into subsets according to their differentiation and activation stages using CD56, CD57 and HLA-DR markers. (B) Surface HLA-DR expression by CD56^bright^ (upper histogram) and CD56^dim^ (lower histogram) NK cells. Fluorescence minus one (FMO) controls of HLA-DR staining are presented. (C) Analysis of cell subset purity after sorting in the single cell mode. (D) The initial proportions of the discriminated subsets (CD56^bright^HLA-DR^–^, CD56^bright^HLA-DR^+^, CD56^dim^CD57^–^HLA-DR^–^, CD56^dim^CD57^–^HLA-DR^+^, and CD56^dim^CD57^bright^) in total NK cells before sorting of donors whose NK cells were used for clone generation. (E) Clone generation frequencies in the subpopulations, mentioned above, determined in clone collections obtained from six donors. (F) Clone generation frequencies in CD56^bright^CD57^–^, CD56^dim^CD57^–^, CD56^dim^CD57^bright^ subsets, recalculated according to the original proportions of HLA-DR^+^ and HLA-DR^−^cells in the subsets. Hereinafter significant differences are shown by asterisks as *p < 0.05; **p < 0.01; ***p < 0.005; ****p < 0.0001.

NK cells circulating in the peripheral blood may differ in their ability to respond to the proposed stimuli, e.g. IL-2 and K562-mbIL21. We hypothesized that differentiation stage of NK cells in each studied individual would affect the proliferative response and frequency of clone formation. To test this, we analyzed individual cell expansion potential by FACS-sorting of single NK cells isolated from several healthy donors according to their maturation stage: CD56^bright^, CD56^dim^CD57^–^ and CD56^dim^CD57^bright^ (Fig 1). The CD56^bright^CD57^–^ subset represented the less mature NK cells and the CD56^dim^CD57^bright^ subset referred to the most differentiated ones [1,18]. To discriminate activated NK cells we have used HLA-DR gating, thus selecting for cloning HLA-DR^−^and HLA-DR^+^ subsets in CD56^bright^ and CD56^dim^CD57^–^ subpopulations (Fig 1A–1C). CD56^dim^CD57^bright^ cells were not divided into HLA-DR^+^ and HLA-DR^−^because of low levels of HLA-DR expression in this subset (1.19 ± 0.52%). Proportions of the selected subsets in circulating NK cells are shown on the Fig 1D. In total, 1093 clonally expanded populations were obtained in six independent collections. The lowest frequency of clone formation was observed in highly differentiated CD56^dim^CD57^bright^ NK cells (Fig 1E and 1F). Despite the large variability between clones from different donors, in each collection cloning efficiency increased by the following scheme: CD57^bright^<CD56^dim^CD57^–^<CD56^bright^ NK cells (Fig 1F). This indicates that larger portions of CD56^bright^ and CD56^dim^CD57^–^ NK cells, compared to more differentiated CD56^dim^ and CD57^bright^ subsets, respectively, start proliferating in response to the IL-2/K562-mbIL21 stimulation (Fig 1F). Therefore, the differentiation stage of NK cells determined their proliferative response in current stimulation conditions. The activation state of NK cell evaluated by HLA-DR expression seemed to have no influence on this process (Fig 1E). Hence, varying single cell expansion rates observed for NK cells from individual donors [9] may, at least in part, result from the differences in the initial proportions of NK cells at certain differentiation stage.

### CD56^dim^CD57^–^HLA-DR^–^NK cells produced most of the long-living clones

**Fig 2:**
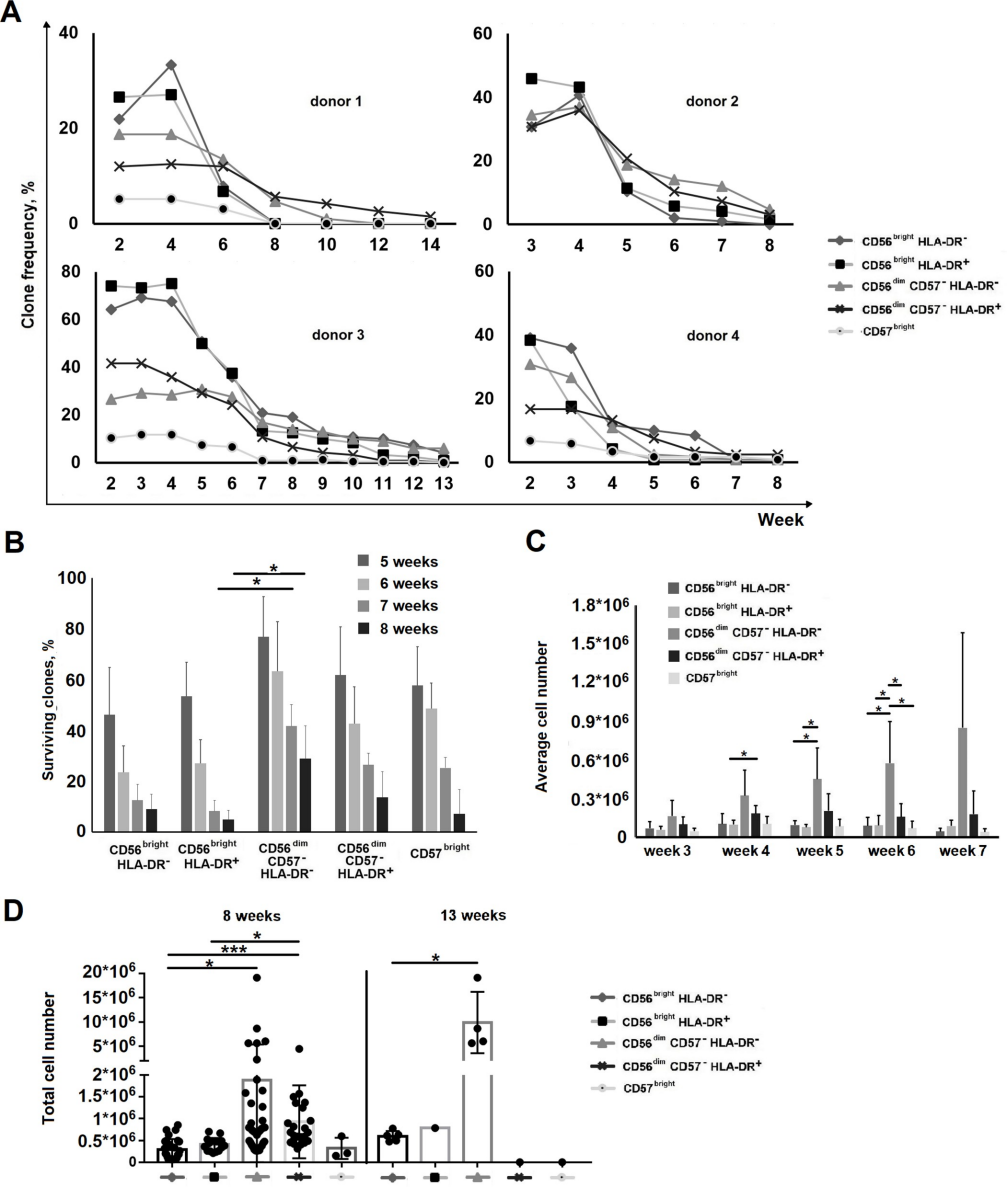
Growing and survival of the NK cell clones. (A) Dynamics of frequency of living clones derived from NK cell subpopulations of various differentiation and activation states. Data of clone collections from four donors are presented. (B) Percentage of survived clones from the subsets, measured at week 5–8 after the beginning of cultivation. Values are Mean ± SE of four clone collections. (C) Dynamics of cell number in clones derived from different subsets. Data averaged on four clone collections are presented as Mean ± SD. (D) Total cell number registered in long-lived clones (8 weeks and 13 weeks) derived from different subsets.

We assessed the lifespan of clones from different subpopulations by weekly counts of clone numbers during cultivation. It was mentioned above that in response to stimulation with IL-2 and K562-mbIL21 CD56^bright^ NK cells formed more clones within 2–4 week time period than CD56^dim^ NK cells (Fig 1E and 1F and Fig 2A). Number of clones decreased dramatically after 5–6 weeks of cultivation. Nevertheless, some individual clones lived for 8 and even 13–14 weeks (Fig 2). More than 80% of clones from CD56^bright^ subset died after 7–8 weeks (Fig 2B). CD56^dim^ NK cells formed fewer clones compared to CD56^bright^ NK cells, however clones from CD56^dim^CD57^–^ subset demonstrated better survival at later time points. Integrally, there was a tendency that clones originating from HLA-DR^−^cells lived longer than from HLA-DR^+^ ones, with the highest percentage of long-lived clones in CD56^dim^CD57^–^HLA-DR^−^subset (Fig 2B and 2E). The lowest level of survival at 7–8 weeks of cultivation was observed in most of clones from CD56^bright^HLA-DR^+^ subset, which was significantly different from the clones derived from CD56^dim^CD57^–^HLA-DR^−^NK cells (Fig 2B).

Along with clone lifespan, a large variability in cell number per clone was observed between different clone collections (Fig 2C). Besides, cell numbers varied greatly within individual clone collection in each studied subset (S1 Fig). However, on average, clones derived from the CD56^dim^CD57^–^HLA-DR^−^subset proliferated better and had more cells during cultivation compared to other clones, whereas the clones derived from other subsets did not increase cell count starting from weeks 4–5 and further, or even significantly decreased up to week 8 (Fig 2C). Several clones with the highest lifespan (13–14 weeks) and the highest total cell number (1–2×10^7^ cells) obtained in this study were generated from the CD56^dim^CD57^–^HLA-DR^−^subset (Fig 2D). At the same time, many clones derived from the CD56^dim^CD57^–^HLA-DR^−^subpopulation were loosing cells after 6 weeks of cultivation. There was no direct dependence of the lifespan on total cell number per clone, which indicates that both cell proliferation and viability contributed to the clone lifespan. Only small portion of NK cells related mostly to CD56^dim^CD57^–^HLA-DR^−^subset was able to respond to the stimulation by long proliferation.

### Assessment of NK cell phenotype stability during clone cultivation

**Fig 3:**
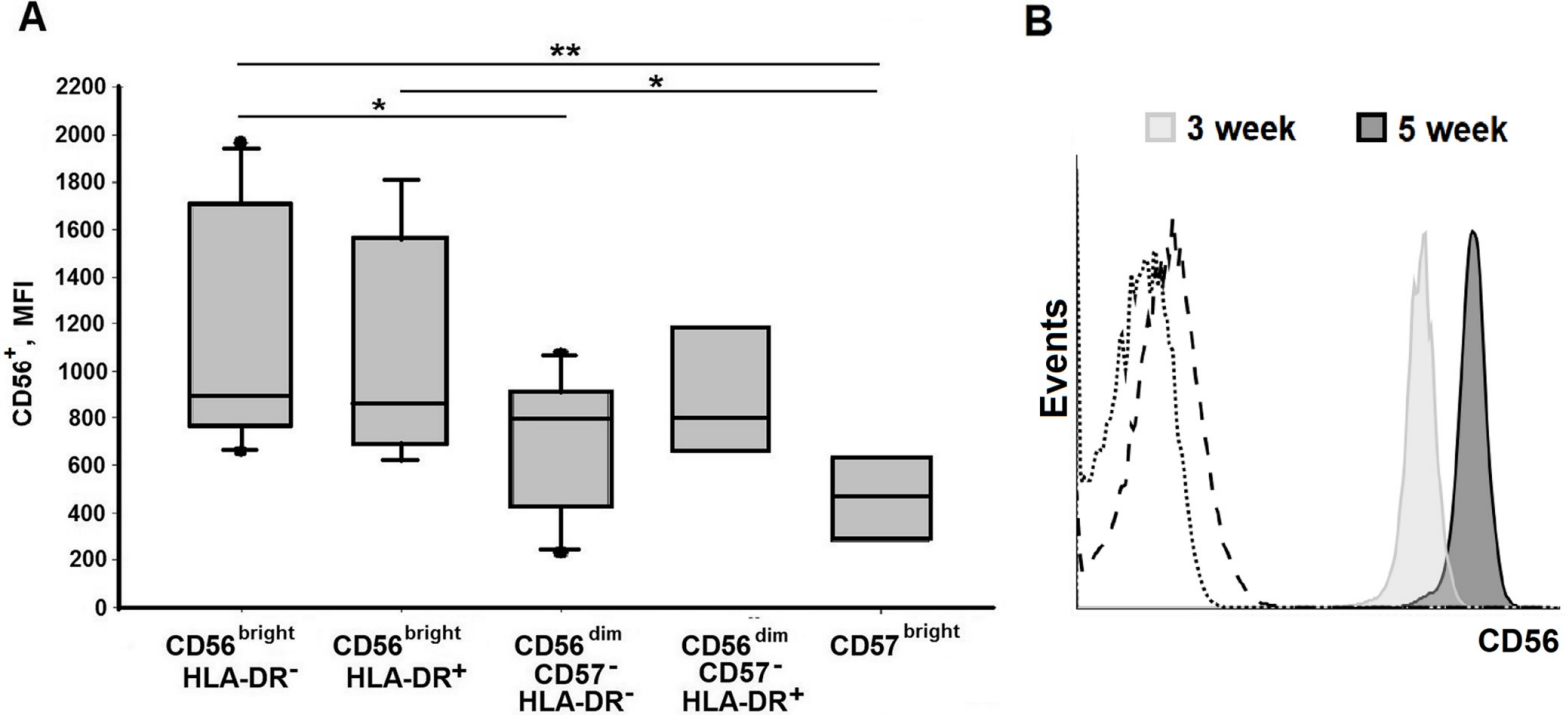
Expression of CD56 in NK cell clones. (A) The expression level of CD56 in NK cell clones obtained from different subsets analyzed 5 weeks after the beginning of cultivation (MFI mean ± SE). (B) CD56 surface expression in a NK cell clone from CD56^dim^HLA-DR^−^subset measured at weeks 3 and 5 of cultivation. The dotted line shows autofluorescence, dashed line is the isotype control.

To estimate the stability of NK cell phenotype during clonal expansion, we studied expression of NK cell markers determining the initial division into subsets, CD56, CD57 and HLA-DR, in 99 clones (21 from CD56^bright^HLA-DR^–^, 20 from CD56^bright^HLA-DR^+^, 20 from CD56^dim^CD57^–^HLA-DR^–^, 21 from CD56^dim^CD57^–^HLA-DR^+^, and 18 from CD56^dim^CD57^+^ subsets) in four clone collections. First, we determined the maturation stage of cells in each clone according to CD56 and CD57 surface levels. CD56 expression level in the clones obtained from the CD56^bright^ subsets remained slightly higher than in the clones obtained from the CD56^dim^ subsets. Generally, CD56 expression level in individual clones increased during five weeks of cultivation (Fig 3B). So, the CD56 level can hardly be used as a marker of differentiation in activated cells. No significant difference was registered between clones from CD56^dim^CD57^–^ and CD56^dim^CD57^bright^ subsets (Fig 3A).

**Fig 4:**
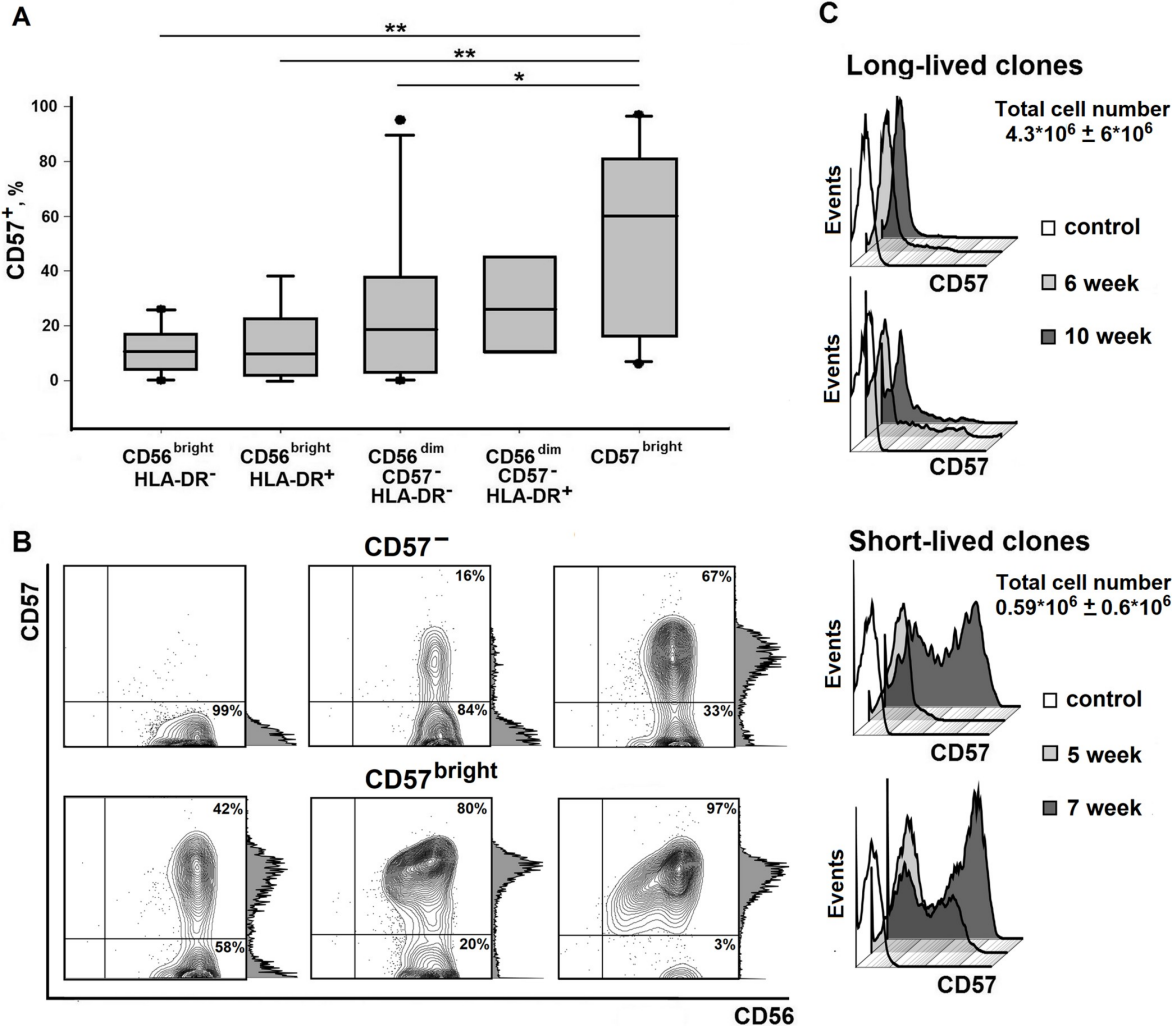
Expression of CD57 in NK cell clones. (A) The expression level of CD57 in NK cell clones derived from different subsets, analyzed 5 weeks after the beginning of cultivation (MFI mean ± SE). (B) Proportion of CD57^+^ cells in NK cell clones from the same donor, obtained from CD57-negative and CD57-positive subsets. (С) Dynamics of CD57 surface expression in short-lived and long-lived clones. Two representative clones for each group are shown. Data on total cell numbers in short-lived (from 5 to 7 weeks) and long-lived (8 weeks and over) clones are presented.

Next, we evaluated CD57 expression in well proliferating clones obtained from different subpopulations (Fig 4). CD57 expression was not clone-specific, and proportion of CD57-positive cells within each clone varied in a wide range (from 0 to nearly 100%) (Fig 4B and 4C). After 5 weeks of cultivation, the clones obtained from CD57-negative CD56^bright^ and CD56^dim^ subsets began to express CD57 on their cell surface, although the average percentage of CD57^+^ cells per clone was significantly lower than for the CD57^bright^-derived clones (Fig 4A). On the other hand, in approximately 30% of analyzed clones derived from CD57^+^ NK cells, the proportion of CD57-positive cells was below 50%. Thus, after 5 weeks of cultivation, part of the CD57^+^ cell’s progeny lost CD57 expression in these clones.

Throughout cultivation, we observed an increase in CD57 expression level at a certain time point in the clones. In the long-lived clones, which had in general higher total cell number, the proportion of CD57-positive cells measured at week 5–6 was lower compared to the short-lived clones, and usually increased during further cultivation (Fig 4C). At the same time, there was no clear association between CD57 expression and viability of clones, since well-proliferating clones with high CD57 expression were observed in certain collections.

**Fig 5:**
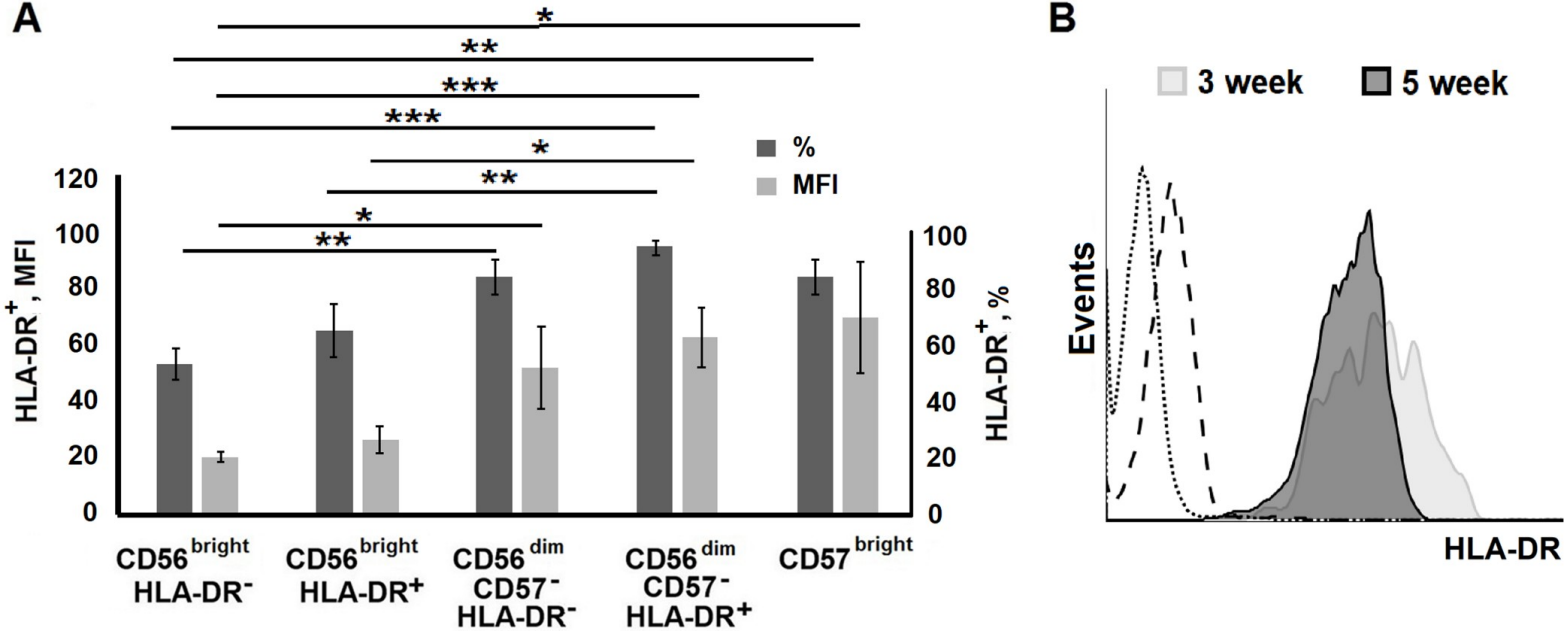
Surface expression of HLA-DR in NK cell clones. (A) HLA-DR expression levels in clones derived from different NK cell subpopulations analyzed at week 5 of cultivation. Proportions of HLA-DR^+^ cells and MFI of HLA-DR^+^ cells (mean ± SE) of n independent experiments are presented: n = 12 for CD56^bright^HLA-DR^–^, n = 9 for CD56^bright^HLA-DR^+^, n = 10 for CD56^dim^CD57^–^HLA-DR^–^, n = 13 for CD56^dim^CD57^–^HLA-DR^+^ and n = 11 for CD56^dim^CD57^bright^. (B) HLA-DR surface expression in a NK cell clone from CD56^dim^HLA-DR^+^ subset measured at weeks 3 and 5. The dotted line indicates autofluorescence, dashed line is the isotype control.

The overall expression of HLA-DR among the clones appears to be one of the main characteristics of the NK cells responding to stimulation with IL-2/K562-mbIL21, although expression levels might differ (Fig 5A and 5B). Differences between clones derived from HLA-DR-positive and HLA-DR-negative NK cells in HLA-DR expression levels were low, which indicated that initially negative cells acquired surface HLA-DR. However, the level of HLA-DR expression was higher in the clones derived from CD56^dim^ cells, compared to the clones from CD56^bright^ cells. Thus, more differentiated cells demonstrated more activated phenotype in response to this type of stimulation. Interestingly, the increase in HLA-DR expression throughout cultivation was registered in clones from CD56^dim^HLA-DR^−^progenitors that gave the longest-lived clones. The most pronounced elevation in HLA-DR expression level was observed in clones derived from CD56^dim^CD57^–^HLA-DR^+^ CD57^bright^ subset (Fig 5).

**Fig 6:**
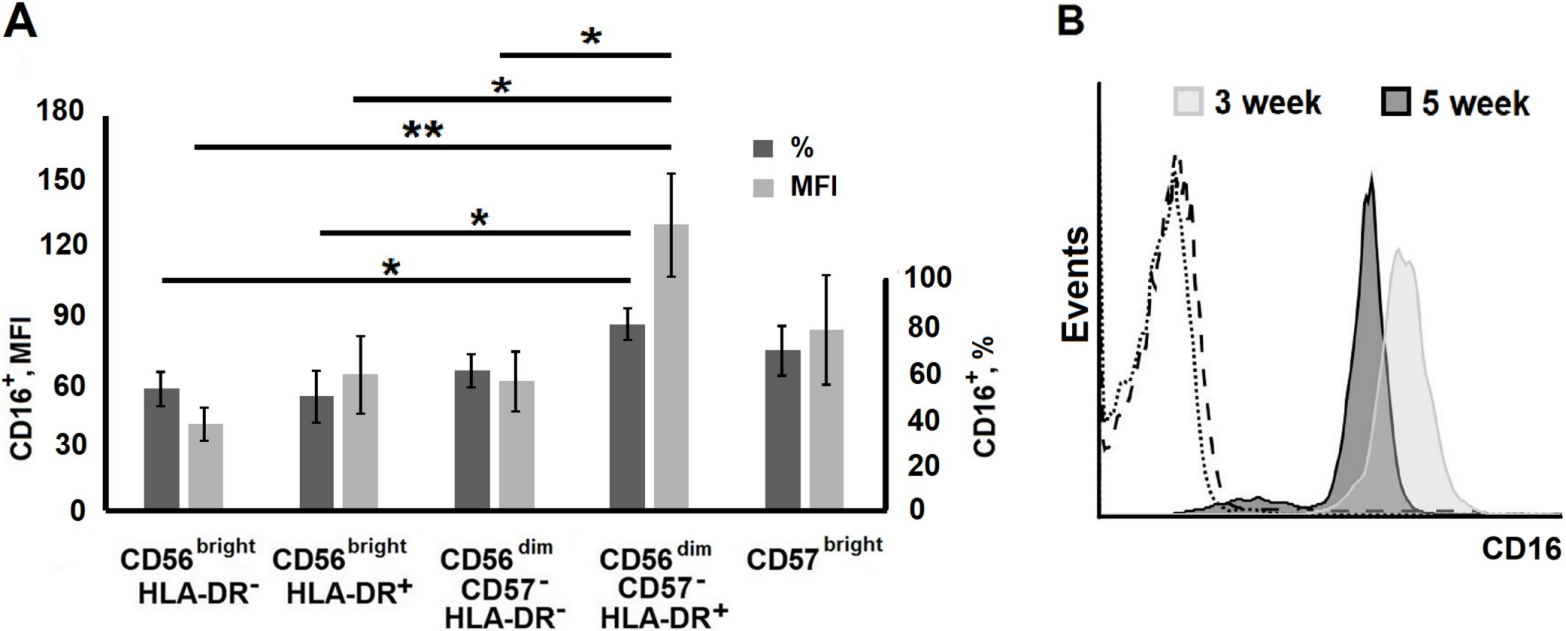
Surface expression of CD16 in NK cell clones. (A) Expression levels of CD16 in clones derived from different NK cell subsets analyzed at week 5 of cultivation. Proportions of CD16^+^ cells and MFI of CD16^+^ cells (mean ± SE) of n experiments are presented: n = 12 for CD56^bright^HLA-DR^–^, n = 10 for CD56^bright^HLA-DR^+^, n = 13 for CD56^dim^CD57^–^ HLA-DR^–^, n = 13 for CD56^dim^CD57^–^HLA-DR^+^, and n = 8 for CD56^dim^CD57^brigh^. (B) CD16 surface expression in a NK cell clone from CD56^dim^HLA-DR^−^subset measured at weeks 3 and 5. The dotted line indicates autofluorescence, dashed line is the isotype control.

CD16 expression was tested in a number of clones. Most of the clones, including initially CD16-negative clones derived from CD56^bright^ subset, expressed CD16. However, CD16 expression level in individual clones varied greatly, which made difficult to detect significant differences between groups. Higher expression of CD16 was observed for HLA-DR^+^ progenitors from CD56^dim^ cell subset compared to clones from HLA-DR^−^progenitors (Fig 6A). It is worth to add that the CD16 expression level usually decreased during clone cultivation (Fig 6B).

### Expression of KIR2DL2/DL3 was clone-specific

**Fig 7:**
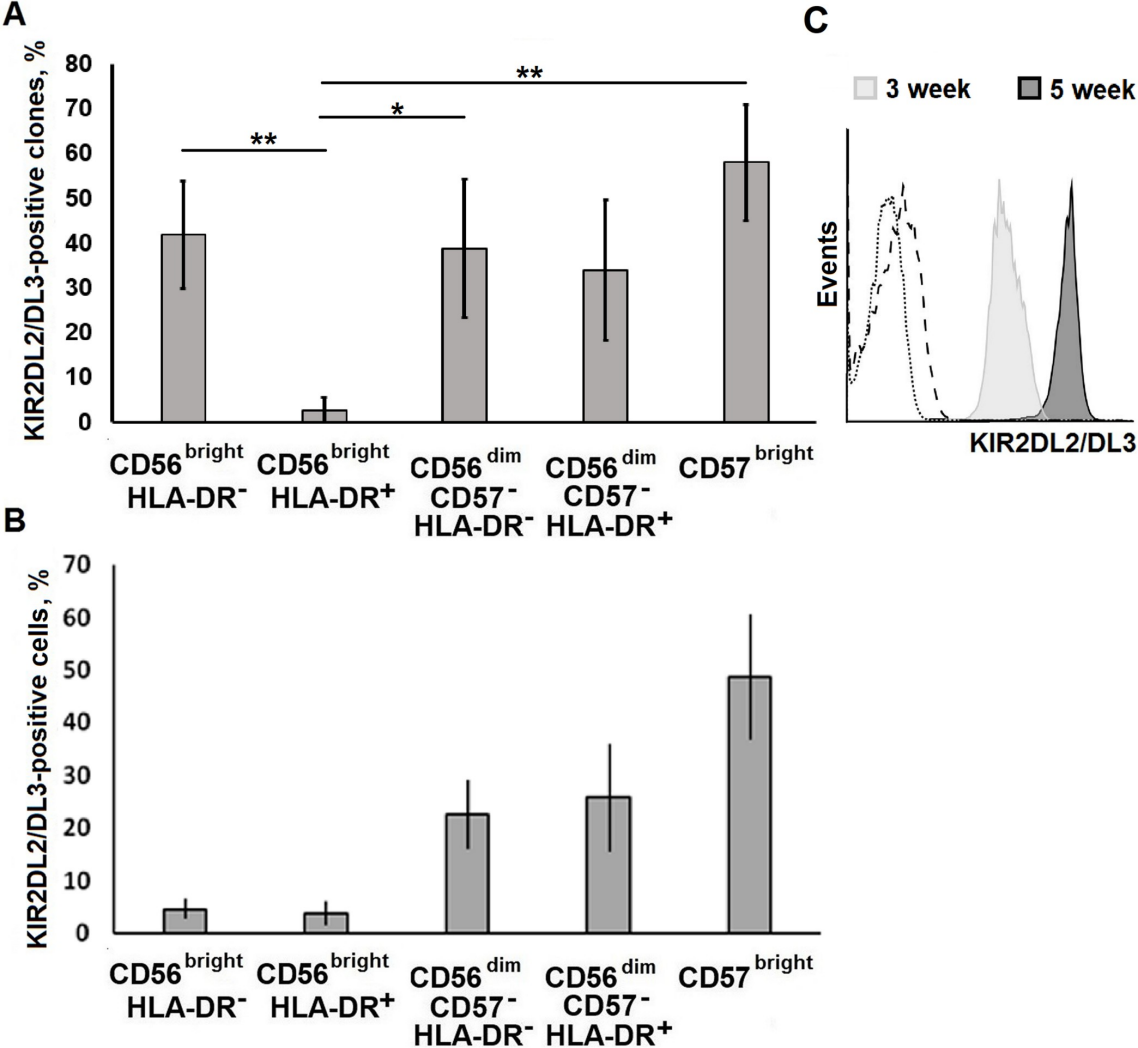
Expression of KIR2DL2/DL3 in NK cell clones. (A) Analysis of KIR2DL2/DL3 expression in clones obtained from different subsets, analyzed after 5 weeks of cultivation. In each subset, the proportion of KIR-positive clones (mean ± SE of 5 independent collections) is presented. In total, KIR2DL2/DL3 expression was analyzed in 139 clones (32 for CD56^bright^HLA-DR^–^, 33 for CD56^bright^HLA-DR^+^, 24 for CD56^dim^CD57^–^HLA-DR^–^, 25 for CD56^dim^CD57^–^HLA-DR^+^, and 25 for CD56^dim^CD57^brigh^ cells). (B) Proportions of the subsets in NK cells *ex vivo*. Mean ± SD of 9 independent experiments is presented. (C) Dynamics of KIR2DL2/DL3 surface expression in an individual NK cell clone. The dotted line indicates autofluorescence, dashed line is the isotype control.

We have analyzed expression of KIR receptors by NK cell clones and freshly isolated NK cells (Fig 7). For immunolabeling we used the antibody specific for the allelic forms KIR2DL2 and KIR2DL3, which are widespread in the human population [19]. Four donors of NK cells participating in the study were heterozygous on HLA-C; each of them possessed one C1 allele recognized by these receptors. The resulting clones were either completely KIR-positive or KIR-negative. At that, surface KIRs never disappeared during cultivation in the positive clones, and never appeared in the negative ones. In KIR-positive clones, the expression level of KIR2DL2/DL3 generally increased during the cultivation process (Fig 7C). The distribution of KIR2DL2/DL3-expressing clones among the studied NK cell subsets mainly reflected that of KIR2DL2/DL3^+^ cells among total NK cells *ex vivo*, with the exception of clones derived from CD56^bright^HLA-DR^−^subset (Fig 7A and 7B). The highest proportion of KIR^+^ clones was found in clone sets obtained from CD57^+^ NK cells. Lower percentages of KIR-positive clones were observed among clones derived from CD57^–^ cells. Surprisingly high proportion of NK cells expressing KIR2DL2/DL3 in clone sets derived from CD56^bright^HLA-DR^−^cells could be explained by better proliferation and viability of KIR^+^ NK cells in these culture conditions. We found no significant differences between the KIR-positive and KIR-negative clones in the proportion of CD57^+^ cells. Thus, there was no clear association between expression of KIR2DL2/DL3 and CD57 in NK cell clones.

### Most of clones grown retained the NKG2A expression or started to express this marker *de novo*

**Fig 8:**
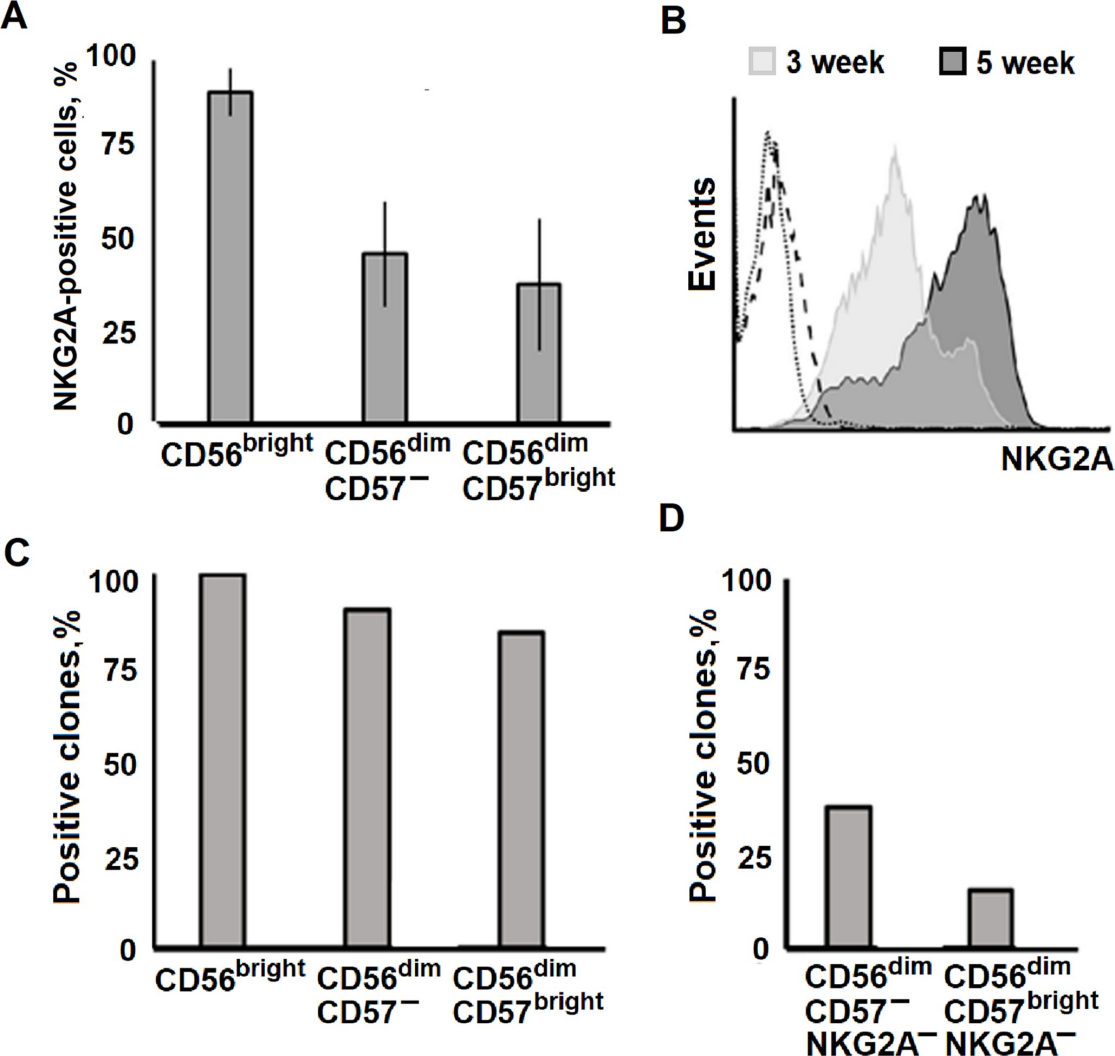
Surface expression of NKG2A in NK cell clones. (A) Proportions of CD56^bright^, CD56^dim^CD57^–^, and CD56^dim^CD57^bright^ subsets in NK cells *ex vivo*. Mean ± SD of 8 independent experiments is presented. (B) Dynamics of NKG2A surface expression in a NK cell clone from CD56^bright^HLA-DR^+^ subset. The dotted line indicates autofluorescence, dashed line–isotype. (C) Analysis of NKG2A expression in clones obtained from different subsets, analyzed after 5 weeks of cultivation. In each subset, the proportion of NKG2A-positive clones is presented (mean ± SE of n clones obtained in 4 clone collections: n = 30 for CD56^bright^, n = 31 for CD56^dim^CD57^–^, n = 19 for CD56^dim^CD57^bright^ subsets). (D) Analysis of NKG2A expression in clones obtained from CD56^dim^CD57^–/bright^NKG2A^–^ subsets, carried out after 5 weeks of cultivation. The proportions of NKG2A-positive clones are presented (mean ± SE of n independent experiments: n = 21 for CD56^dim^CD57^–^NKG2A^–^, n = 19 for CD56^dim^CD57^brigh^NKG2A^–^ subsets).

In freshly isolated peripheral blood NK cells, nearly all CD56^bright^ cells were positive for NKG2A (Fig 8A), a lectin-like inhibitory receptor, which characterizes less mature NK cells [5]. In their turn, only 50% (approximately) of CD56^dim^ NK cells expressed NKG2A (Fig 8A). Analysis of NKG2A expression in clones conducted in this work has revealed the similar pattern of distribution of NKG2A-expressing clones. Most of the clones from all NK cell subsets, analyzed at different time points throughout their cultivation, were positive for NKG2A. Of the 49 clones analyzed, 45 clones expressed NKG2A (more than 90% of clones), and only 4 clones (all derived from CD56^dim^ NK cells) were NKG2A-negative (Fig 8B and 8C). To reveal whether NKG2A-negative NK cells acquire NKG2A expression during clonal expansion, CD56^dim^NKG2A^–^ NK cells were cloned. Phenotype analysis of the obtained clones was performed in five weeks. A part of clones from both CD56^dim^CD57^–^ and CD56^dim^CD57^bright^ subsets expressed NKG2A (Fig 8D). This result indicates the ability of mature NKG2A^–^ NK cells to express NKG2A *de novo* in specific stimulating conditions. However, a part of clones remained NKG2A negative, which points out that expression of NKG2A is not strictly associated with successful clonal expansion.

### Acquired NK cell clones demonstrated functional activity

**Fig 9:**
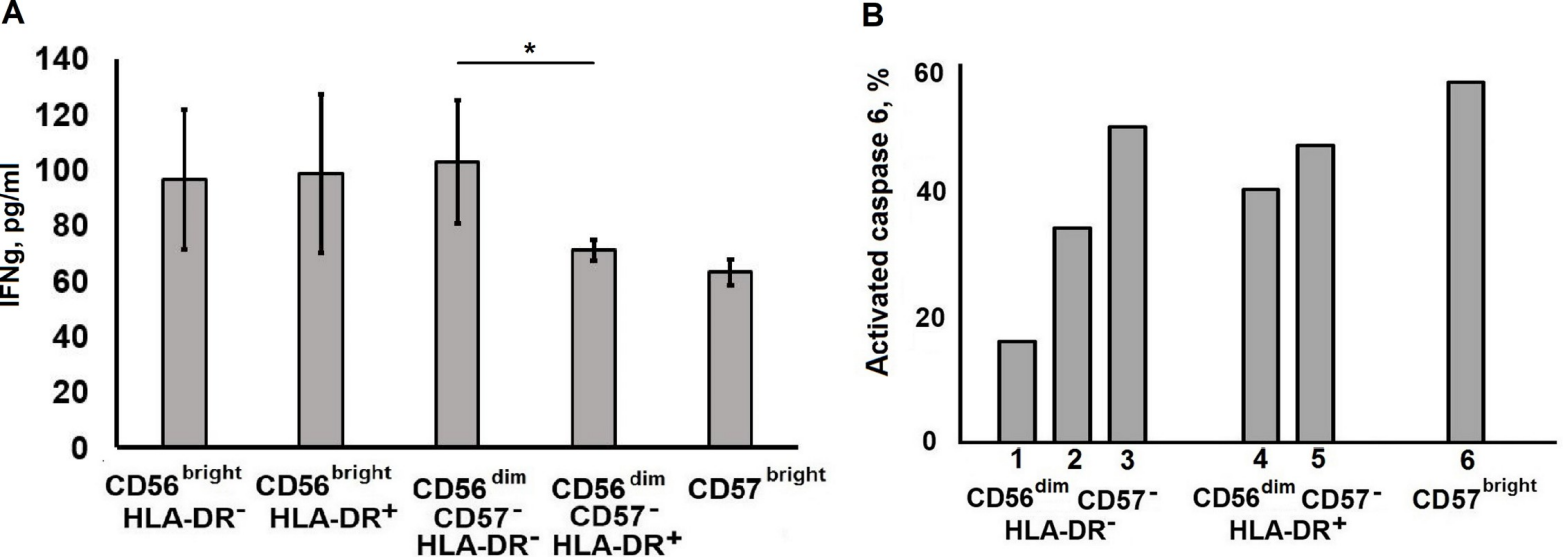
Functional activity of NK cell clones. (A) IFN-γ production was analyzed by ELISA in 25 clones from one collection: 4 for CD56^bright^HLA-DR^–^, 4 for CD56^bright^HLA-DR^+^, 4 for CD56^dim^CD57^–^HLA-DR^–^, 9 for CD56^dim^CD57^–^HLA-DR^+^, and 4 for CD56^dim^CD57^brigh^ cells. (B) Natural cytotoxic activity of clones derived from different NK cell subsets.

We have analyzed in short screen the ability of NK cell clones to produce cytokines. All analyzed clones produced IFNγ in response to IL-12+IL-15 stimulation. Remarkably, significant difference in IFNγ levels was found between clones derived from CD56^dim^CD57^–^HLA-DR^−^and CD56^dim^CD57^–^HLA-DR^+^ subsets with higher levels in HLA-DR-negative subpopulation (Fig 9A). In several well-growing clones, all originated from CD56^dim^ NK cells, natural cytotoxicity levels against K562 cells was assessed at week 6 of cultivation. All tested clones demonstrated considerable cytotoxic activity (Fig 9B). Thus, NK cell clones developed in the IL-2/K562-mbIL21 stimulation system were functionally active.

## Discussion

A variety of stimulation methods can be applied to activate and expand NK cells [9,20,21]. Some of them can be used to obtain NK cell clones [22,23]. Most of the stimulation approaches are based on combinations of cytokines acting cooperatively on NK cells. Along with IL-2, IL-21 has been shown to enhance the antitumor properties of NK cells [24]. In this work the stimulation method including the usage of IL-2 together with membrane-bound IL-21 expressed on K562 cells was applied to generate NK cell clones. This combination was shown to induce extensive proliferation of NK cells in earlier study [9]. We have described phenotypic characteristics of clones generated from NK cell subpopulations of various differentiation and activation stages and have shown that, surprisingly, NK cells are able to acquire less mature phenotype, according to CD57 and NKG2A expression levels, in specific conditions.

It was shown previously NK cells from CD56^dim^ and CD56^bright^ subsets differ in functional response and signaling when grown in the presence of IL-2 and IL-21 [25]. The state of the responding cells, such as the repertoire of expressed receptors and intracellular signal-transducing unit ratios, determines their proliferative and functional activity. The subset of highly differentiated CD57^+^ NK cells, which increases proportionally with age and in the presence of certain chronic infections [18], can also respond to the stimulation by its own way. Varying efficacy of the response to IL-2/K562-mbIL21 of NK cells at distinct differentiation stages may to some extent mediate the variations in expansion rate of NK cells between donors with different ratio of more or less differentiated cells. In this work, we have examined CD56^bright^, CD56^dim^CD57^–^, and CD57^bright^ NK cells for their ability to produce clones. We have revealed several differences between these subpopulations in terms of the effectiveness of clone generation, clone life-span and phenotype. The frequency of clone formation was the highest in the CD56^bright^ subpopulation. This is likely due to the fact that CD56^bright^ cells have a high affinity receptor for IL-2, which means they respond better to the stimulation [5]. It is important to note that in six donors studied this frequency varied significantly, from 28% in donor 1 to 70% in donor 3 (Fig 1E and 1F). The low reproducibility in the clone formation could be partially explained by unaccounted variations in plating efficiency in different experiments. It is also possible, that other factors independent of NK cell differentiation and activation state could also affect the effectiveness of cloning.

Another important parameter examined here was the lifespan of clones, including proliferative activity and survival of clonal cells. Interestingly, the CD56^bright^ subsets characterized by a better ability to generate clones, showed in general poor survival during cultivation. The longest-lived clones were mostly derived from the CD56^dim^CD57^–^ subset, with maximal total cell number in clones derived from HLA-DR^−^cells, which were not in activated state (Fig 2). This could be attributed to the preferable response of CD56^dim^ NK cells to K562 and membrane-bound IL-21. Interestingly, IFN-γ production was significantly higher in clones from CD56^dim^CD57^–^HLA-DR^−^cells compared to CD56^dim^CD57^–^HLA-DR^+^ (Fig 9A).

In addition the clones from CD56^dim^ subset demonstrated higher expression of HLA-DR than clones from CD56^bright^ cells (Fig 5). In earlier work a higher expression level of CD69, another marker of cell activation, was observed during IL-21-induced NK-cell proliferation in CD56^dim^ subpopulation compared to CD56^bright^ [25]. It should be noted that, on average, the lifespan exceeded 8 weeks only in less than 30% of clones from CD56^dim^CD57^–^ subset. Surviving clones were usually characterized by higher expansion. The reasons for the higher lifespan of such clones from CD56^dim^CD57^–^ subset compared to the clones from CD56^bright^ subset are still unknown.

In NK cell clones originated from CD57^–^ subsets a part of cells acquire CD57 expression during cultivation. It may be explained by “senescence” of NK cell culture. Proportion of CD57^+^ cells in such clones in most cases inversely correlated with clone longevity. This observation corresponds well with the previously described results evidencing that CD57 acquisition by NK cells leads to lower proliferation ability [26].

Interestingly, a significant proportion of clones (about 30%) derived from the CD57^bright^ subset contained cells negative for surface expression of CD57 (Fig 4A and 4B). This observation indicates that in certain conditions NK cells can lose CD57 expression during clonal expansion. According to current view [1] acquisition of CD57 expression by NK cells is an irreversible step of differentiation. This discrepancy may be explained by different experimental conditions used in these works. Various CD57 expression levels among clones may reflect the differentiation stage or functional state of the clone cells.

An overwhelming majority of the clones expressed the lectin-like NKG2A receptor, which is typical for less differentiated NK cells. This corresponded to earlier data showing that the incubation of NK cells with IL-21 resulted in both an increase in the number of NKG2A-positive NK cells and the expression of the *NKG2A* gene [27]. Obviously, IL-21 did not induce differentiation of NK cells, but rather promoted the expansion of cells with less mature phenotype. High proportion of NKG2A-expressing NK cells (about 70%) was earlier observed in NK cell cultures stimulated with IL-2 / K562-mbIL2. In the same cultures, longer telomeres were found in the expanded NK cells [9]. We have shown that NKG2A-negative NK cells, even that expressed CD57, may in response to this stimulation start to express NKG2A *de novo*. This may be linked to the loss (at least partial) of surface CD57. At the same time, it should be noted that in the absence of NKG2A expression NK cells are able to form clones (Fig 8D). So, NK cell proliferation does not necessarily require NKG2A expression.

Bjorkstrom and coworkers showed that surface expression of various KIR receptors increases with differentiation of NK cells reaching a maximum in CD57^bright^ subset [1]. Distribution of KIR2DL2/DL3 between the NK cell clones (Fig 7) mainly corresponded to the pattern described in this work. Identical pattern of KIR expression within an individual clone (all cells either negative or positive) confirms that, in general, KIR expression is highly conservative and maintains during cultivation. However, clones developed from CD56^bright^HLA-DR^−^(which represents a small subset of peripheral blood NK cells) seem to be an exception: the proportion of KIR2DL2/DL3^+^ clones in this set was much higher than in CD56^bright^HLA-DR^+^-derived clone set and was comparable to the proportion in CD57^bright^-derived clones. Greaten proliferation response of CD56^bright^HLA-DR^−^NK cells to the stimulation with IL-2 / K562-mbIL21 or/and increased viability of resulting clones can be considered as most possible reasons for it, although *de novo* expression of KIR molecules can not be formally excluded [1]. Since the NK cell donors were heterozygous on HLA-C and possessed both C1 and C2 alleles, it can not be excluded that the advantage in clonal expansion of the KIR^+^ NK cells from CD56^bright^HLA-DR^−^subset might be connected to the interaction of KIR2DL2/DL3 receptors with the HLA-C1 ligands exposed on neighboring NK cells.

We intended to examine the effectiveness of clone generation by *ex vivo* HLA-DR^+^ NK cells in response to IL-2/K562-mbIL21 stimulation. It is known that HLA-DR-positive cells constitute only a small fraction of NK cells of healthy people, although their proportion increases in some pathological conditions associated with chronic inflammation or infection [26,28], multiple sclerosis [6] and IgA-nephropathy [29]. Recently, it has been shown that the level of HLA-DR^+^ NK cells correlated with clinical improvements in tumor patients [30]. These cells may have already undergone activation *in vivo* under the influence of certain stimuli. In the current study, HLA-DR-positive NK cells had no advantage in forming clones upon stimulation with IL-2/K562-mbIL21. Moreover, the lowest percentage of long-lived clones after 7–8 weeks of cultivation was observed in the CD56^bright^HLA-DR^+^ subpopulation (Fig 2C). In the CD56^dim^ subsets, clones derived from HLA-DR^+^ NK cells also died faster. The initial high percentage of circulating HLA-DR-positive NK cells is therefore likely to be a negative factor for the expansion of such cells for clinical use. On the other hand, clones derived from HLA-DR-positive cells tended to have a higher expression level of CD16 (Fig 6). After 3 and all the more 5 weeks of cultivation clones obtained from HLA-DR-negative NK cells started expressing this molecule, and the differences in HLA-DR expression levels between initially HLA-DR-negative and HLA-DR-positive NK cells almost disappeared. The highest HLA-DR level after 5–6 weeks of cultivation was observed in few well-growing clones derived from CD57^bright^ cells. This subpopulation could contain the subset of CD56^bright^NKG2C^+^ adaptive NK cells which, as we have shown earlier, express increased HLA-DR level [31]. Hypothesis that adaptive NK cell clones are growing under stimulation with IL-2/K562-mbIL21 needs to be tested in a future study.

Stimulation with IL-2 and/or IL-21 results in the increase of HLA-DR expression on the surface of NK cells [32–34]. Expression of HLA-DR has been proposed as a marker of NK cell activation. Besides, IL-21 in both soluble and membrane-bound form augments the production of IFN-γ in NK cells [27,35]. In this and earlier works of our group, HLA-DR expression was detected in NK cell clones obtained by combined stimulation with IL-2/K562-mbIL21 [8]. Moreover, this type of stimulation induced HLA-DR expression in cells that were initially negative for this marker (Fig 5A).

According to numerous studies, the natural cytotoxicity of NK cells is increased by IL-21 [25,34,36]. NK cells expanded with stimulation with IL-2 and IL-21 in combination with EBV-LCL feeder cells, exhibited cytotoxicity against a variety of tumor targets [37]. In this study, we did not perform a wide screening of the cytotoxic activity in the obtained clones. Still, in all randomly selected CD56^dim^ clones we observed a significant level of natural cytotoxicity at week 6 of cultivation, which confirmed their functional activity (Fig 9B).

In summary, the reproducible approach for generating NK cell clones described here allowed to define parameters of phenotypic stability and plasticity during the cloning process, and to identify varying cloning efficiency among NK cells at distinct differentiation stages. These results can be useful when choosing a stimulation regimen for NK cell expansion, including cloning, for clinical purposes. Also we have shown for the first time that the loss of CD57 expression by mature CD57^+^ NK cells is basically possible.

## Methods

### Isolation of human NK Cells

Blood samples in this study were derived from healthy individuals of different ages, male and female. All participants gave their verbal informed consent prior to the study that was approved by local ethics committee (Pirogov Russian National Research Medical University). Blood was collected in heparinized tubes, then diluted with PBS (PanEco, Russia) and layered on a standard Ficoll solution (PanEco, Russia) to isolate peripheral blood mononuclear cells (PBMC) using gradient centrifugation. NK cells were obtained from PBMC by negative magnetic separation using a human NK cell isolation kit (Miltenyi Biotech, Germany) according to the manufacturer’s protocol. The purity of NK cells in the preparations after the procedure was no less than 97% based on immunolabeling [38].

### Cell lines

The K562 cell line (human erythroblastoid leukemia) was obtained from ATCC (Manassas, VA, USA). The gene-modified clone of K562 cells (К562-mbIL21) expressed membrane-bound IL-21, CD64, CD86, CD137L (4-1BBL), and truncated CD19 was used [9]. Cells were cultivated in RPMI-1640 medium (PanEco, Russia) containing 10% fetal calf serum (FCS) (HyClone, USA), 2 mM of L-glutamine (PanEco, Russia) and an antibiotic antimycotic solution (Millipore-Sigma, USA) at 2–8×10^5^ cells/ml to maintain cell growth in log-phase. Surface expression of IL-21 during cultivation was tested periodically by flow cytometry using IL-21-PE antibody (BioLegend, USA, clone 3A3-N2). Prior to use as feeder cells, К562-mbIL21 cells were irradiated with γ radiation (100 Gy) and frozen in 90% FCS containing 10% DMSO (Sigma, USA) at –150°C.

### Surface fluorescent immunostaining and flow cytometry

The following anti-human monoclonal antibodies (mAbs) were used for flow cytometry: CD3- PE-Cy7 (Beckman Coulter, USA, clone UCHT1), CD56-APC (Beckman Coulter, USA, clone N901), CD56-Brilliant Violet 421 (Sony, USA, clone HCD56), CD56-PE (Beckman Coulter, USA, clone N901 (HLDA6)), CD57-PE (eBioscience, USA, clone TB01), CD57-FITC (Miltenyi Biotech, Germany, clone TB03), CD57-APC (Miltenyi Biotech, Germany, TB03), CD16-PE (Sony, USA) anti-NKG2A-PE (R&D Systems, USA, clone 131411), anti-KIR2DL2/DL3-PE (Miltenyi Biotech, Germany, clone DX27), anti-HLA-DR-FITC (Beckman Coulter, USA, clone B8.12.2). Cells were incubated in PBS containing 0.5% BSA (bovine serum albumin) and 0.01% sodium azide (labeling buffer) with mAbs for 30 min on ice, then washed twice with labeling buffer and centrifugation. Samples were subsequently analyzed using FACSCalibur flow cytometer (BD Biosciences, San Jose CA, USA) equipped with 488 and 640 nm lasers. At least 30000 events were recorded in lymphocyte gate for total NK cell population and 5000 events for NK cell clones. Acquired data was analyzed using Flowing Software version 2.5.1 (PerttuTerho, Turku Centre for Biotechnology, Finland) and FlowJo software version 7.6 (FlowJo LLC, Ashland, OR, USA). For fluorescence-activated cell sorting, cells were labeled with mAbs in PBS containing 0.5% BSA and 2 mM EDTA.

### Generation of NK cells clones using fluorescence-activated single cell sorting

NK cell clones were obtained accorging to the protocol (dx.doi.org/10.17504/protocols.io.t6teren). Freshly isolated NK cells were immunolabeled with fluorescent-conjugated mAb towards CD56, CD57, CD3 and HLA-DR. Different subpopulations of NK cells (CD56^bright^HLA-DR^–^, CD56^bright^HLA-DR^+^, CD56^dim^CD57^–^HLA-DR^−^or CD56^dim^CD57^–^HLA-DR^+^, CD57^bright^) were then sorted into 96-well round-bottom plates, one cell per well in single cell mode using FACSVantageDiVa cell sorter (BD Biosciences, San Jose, CA, USA) equipped with 405, 488, 643 nm lasers and an appropriate set of detectors and filters. At least 120 wells were seeded with single NK cells from each subpopulation. Prior to sorting, the plates were pre-loaded with 2×10^3^ K562-mbIL21 feeder cells per well in 200 μl of complete medium for clones (DMEM medium (PanEco, Russia) containing 20% ExVivo medium (Thermo Fisher Scientific, Carlsbad, CA, USA) and 100 U/ml of recombinant human IL-2 (Hoffmann-La-Roche, Switzerland)) per well. After 3 weeks incubation (37°C, 5% CO_2_) half of the medium was replaced. After 6 weeks K562-mbIL21 feeder cells (2×10^3^ per well) were added to growing clones.

### Estimation of NK cell clone frequency, cloning efficiency, cell number and life-span

Emergence of NK cell clones in the wells was recorded visually. The percentage of wells with clones among all seeded wells (clone frequency) in CD56^bright^HLA-DR^–^, CD56^bright^HLA-DR^+^, CD56^dim^CD57^–^HLA-DR^–^, CD56^dim^CD57^–^HLA-DR^+^, CD57^bright^ subsets or whole NK cell population was calculated every week beginning from week 2 to week 7–14 of cultivation, depending on the collection. Clone generation efficiency in each subpopulation was measured by determining clone frequency estimated after weeks 2, 3 or 4, when the greatest number of clones was detected. Clone generation efficiency in whole CD56^bright^ or CD56^dim^ subset was calculated basing on the initial frequencies of the CD56^bright(dim)^HLA-DR^+^ (F_i1_) and CD56^bright(dim)^HLA-DR^−^(F_i2_) cells in the whole NK cell population as (F_i1_×F_g1_+F_i2_×F_g2_)/(F_i1_+F_i2_), where F_g1_ –clone generation efficiency of CD56^bright(dim)^HLA-DR^+^, F_g2_ –clone generation efficiency of CD56^bright(dim)^HLA-DR^–^. The percentage of surviving clones in each subpopulation was calculated as the ratio of the number of clones detected on certain week to the maximal number of clones detected on week 2, 3, or 4, dependent on the collection. Clones surviving for 8 or more weeks were considered long-lived. The number of cells in clones was assessed periodically by automated cell counter TC20 (Bio-Rad Laboratories, USA).

### IFN-γ production analysis

10^5^ cells of an individual NK cell clone (at week 5 of cultivation) were transferred to a new 96-well round-bottom plate with fresh complete medium for 24 h, then washed and incubated with IL-12 (10 ng/ml) and IL-15 (10 ng/ml) for 18 h. Supernatants were then collected and IFN-γ level was analyzed by ELISA (Vector-Best, Russia). Optical density was measured using Multiscan FC plate reader (Thermo Fisher Scientific, USA) with 450 nm basic filter and 620 nm reference filter. Concentrations were calculated based on standard curves and formulas provided with the kit.

### Natural cytotoxicity assessment

Natural cytotoxicity was measured using CyToxiLux kit for caspase 6 activation (CTL602, OncoImmunin, USA) used according to the manufacturer’s protocol. Briefly, K562 cells were labeled with the TFL4 tracking dye in RPMI-1640 medium, washed and then added to NK cells in 1:1 proportion. Cells were centrifuged, and supernatants were replaced with 50 ul of caspase 6-specific substrate. The samples were incubated for 30 min, 37°C. Samples were analyzed by flow cytometry, with activated caspase 6 measured in the fluorescein detector and TFL4 in the long red detector. The percentage of target cells with activated caspase 6 was analyzed by the fluorescence of cleaved caspase-6 substrate (fluorescein-positive) in the tracking dye TFL4-positive K562 cell population.

### Statistical analysis

Statistical significance of the differences in data with normal distribution was determined by Student’s t-test. Throughout the text data are presented as mean ± SD unless otherwise specified. For non-normally distributed data a Mann-Whitney U-test was used. P-values of < 0.05 were considered significant.

## Supporting information

S1 Fig Dynamics of cell numbers in clones derived from different subsets. Data of one individual collection are presented as Means ± SD.
(TIF)
