# Supplementary figures and images for: Analysis of NK cell clones obtained using interleukin-2 and gene-modified K562 cells revealed the ability of “senescent” NK cells to lose CD57 expression and start expressing NKG2A
Source: PLoS One. 2018 Dec 5;13(12):e0208469. doi: 10.1371/journal.pone.0208469 (PMC6281266; doi:10.1371/journal.pone.0208469)

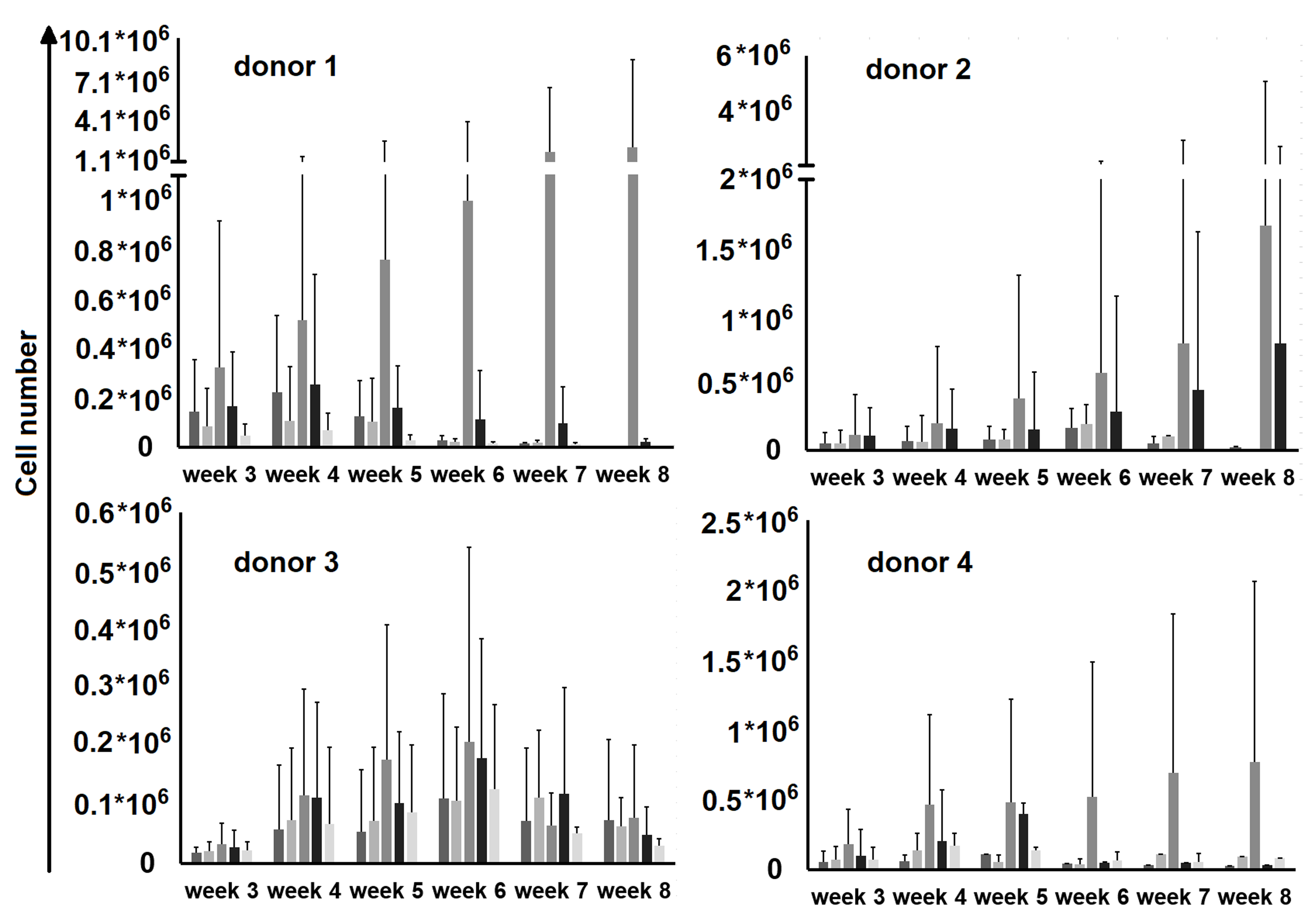

Supplement: S1 Fig — Data of one individual collection are presented as Means ± SD. (TIF) [file pone.0208469.s001.tif]
